# Socio-demographic and behavioural characteristics associated with HSV-2 sero-prevalence in high risk women in KwaZulu-Natal

**DOI:** 10.1186/s13104-015-1093-0

**Published:** 2015-05-05

**Authors:** Nathlee Samantha Abbai, Handan Wand, Gita Ramjee

**Affiliations:** HIV Prevention Research Unit, Medical Research Council, 123 Jan Hofmeyr Road, Westville, Durban, 3630 South Africa; Department of Epidemiology and Population Health, London School of Hygiene and Tropical Medicine, London, UK; The Kirby Institute, University of New South Wales, Sydney, NSW 2052 Australia

**Keywords:** HSV-2, Sero-prevalence, Multivariate analysis, Socio-demographic, Sexual behaviour

## Abstract

**Background:**

The World Health Organization estimates that 536 million people aged 15–49 are infected with Herpes simplex virus type 2 (HSV-2), the causative agent of genital herpes. The aim of this study was to investigate the role of behavioral and demographic factors that contribute to the high HSV-2 sero-prevalence among women participating in a HIV prevention trial. The Methods for Improving Reproductive Health in Africa (MIRA) study assessed the effectiveness the latex diaphragm and lubricant gel on HIV prevention among women in South Africa and Zimbabwe. At screening an interviewer administered questionnaire on demographics and sexual behaviour was obtained. HSV-2 serum antibodies were detected using HerpeSelect™ ELISA IgG. Statistical analysis was performed using STATA release 12.0. This study was registered with ClinicalTrials.gov,number NCT00121459 on the 28th February 2007.

**Findings:**

Of the 3 472 women screened at the Durban research sites 2 218 (73%) had a prevalent HSV-2 infection and 1431 (41%) of the women were also co-infected with HIV. In the multivariate analyses, older women (adjusted odds ratio) [aOR]: 3.49, 95% CI: (2.71,4.49) for >35 years and aOR: 1.82, 95% CI: 1.49, 2.22 for 25–34 years compared with <25 years, p < 0.001 for both comparisons were more likely to be HSV-2 sero-positive. Low level of education (OR: 1.26 95% CI: 1.03, 1.53), having >1 life-time sexual partners (OR: 2.48, 95% CI: 1.92, 3.20), parity >1 (OR: 1.95 95% CI: 1.92, 3.20) and being HIV positive (OR: 6.31, 95% CI: 5.06, 7.88) were significantly associated with HSV-2 infection.

**Conclusion:**

The high sero-prevalence of HSV-2 in the studied population is of great public health importance since this high risk population could act as a reservoir for future infections particularly HIV transmission.

## Findings

While bacterial sexually transmitted infections (STIs) such as Chlamydia and Gonorrhoea are curable, viral STIs such as herpes simplex virus, human papillomavirus, hepatitis B and Human immunodeficiency virus (HIV) are deemed to be persistent and incurable [1,2]. The World Health Organization estimates that 536 million people aged 15–49 are infected with Herpes simplex virus type 2 (HSV-2), the causative agent of genital herpes [3]. Annually approximately 23.6 million people in this age group become newly infected with HSV-2. The prevalence rates of HSV-2 infection are reported to be higher in women when compared to men; with the lowest prevalence rates being 13% among West European men and the highest prevalence rates being 70% among sub-Saharan African women [3]. There are several reports suggesting that HSV-2 is a biological co-factor for HIV acquisition [4-7]. A meta-analysis performed by Freeman [8] showed that the relative risk of HIV acquisition associated with HSV-2 was 3.1 (95% confidence interval (CI): 1.7, 5.6). It is suggested that HSV-2 infection may contribute to >50% new HIV infections among women in Sub-Saharan Africa [9]. In South Africa, the prevalence of HSV-2 infection is reported to be 53% [10].

The aim of this analysis was to investigate the role of behavioural and demographic factors that contribute to the high HSV-2 sero-prevalence among women participating in a HIV prevention trial. The Methods for Improving Reproductive Health in Africa (MIRA) study that was conducted between 2003–2005 assessed the effectiveness the latex diaphragm and lubricant gel on HIV prevention among women in South Africa and Zimbabwe [11]. In Durban, the study was conducted in rural Umkomaas and Bothas Hill, southern and western Durban. The study protocol and informed consents were approved by the Biomedical Research Ethics Committee of the University of KwaZulu-Natal. Participants provided written informed consent prior to study procedures being conducted.

At screening, verbal consent to assess initial eligibility, followed by written informed consent for screening procedures, including diagnostic testing and answering an interviewer administered questionnaire on demographics and sexual behaviour was obtained. The full details of this study are described elsewhere [11]. The presence of HSV-2 antibodies was detected using HerpeSelect™ ELISA IgG (Focus Technologies, Cypress, CA, USA) according to the manufacturer’s recommendations with a cut-off value of 1.1. Two HIV rapid tests were done on whole blood samples from venipuncture by use of Determine HIV-1/2 (Abbott Laboratories, Japan) and Oraquick (OraSure Technologies, USA). Urine specimens were collected for polymerase chain reaction (PCR) testing for *Neisseria gonorrhea* (NG), *Chlamydia trachomatis* (CT) and *Trichomonas vaginalis* (TV) (Roche Pharmaceuticals, USA). Univariate and multivariate analyses were performed using logistic regression for both. Multivariate models considered all variables statistically significant (*P* < 0.10) in the initial analyses and used a forward stepwise method. Statistical analysis was performed using STATA release 12.0 (College Station, Texas, TX, USA).

Of the 3 472 women screened at the Durban research sites 2 218 (73%) had a prevalent HSV-2 infection (Table 1). According to the univariate analyses, socio-demographic associations such as being older (25 years and older) (Odds Ratio (OR) 2.37, 95% Confidence Interval (CI): 2.00,2.81), belonging to a particular ethnic group based on language spoken i.e. isiZulu (OR: 5.47, 95% CI: 4.01, 7.43) having less than high school education (OR: 1.68, 95% CI: 1.43, 1.98), being single/non-cohabiting (OR: 1.54, 95% CI: 1.25, 1.89), and belonging to a particular religion (Christianity) (OR: 1.72, 95% CI: 1.36, 2.18) were significantly associated with HSV-2 infection. The behavioural and biological factors that were significantly associated with HSV-2 infection were: age of sexual debut (<16 years old) (OR: 1.40, 95% CI: 1.19, 1.63), >4 lifetime sexual partner (OR: 5.43 95% CI: 4.33, 6.82) parity >1 (OR: 2.31, 95% CI: 1.90,2.80), lack of contraceptive use (OR: 1.40, 95% CI: 1.26, 1.68), co-infection with *Neisseria gonorrhoeae* (OR: 2.28, 95% CI: 1.29, 4.05) presenting with a genital ulcer (OR: 3.16, 95% CI: 1.73, 5.74) and being HIV positive (OR: 7.38, 95% CI: 6.01, 9.10).

**Table 1:** **Socio-demographic, behavioural and biological data associated with Herpes Simplex Virus type −2 (HSV-2) in women that presented for screening**

**HSV-2 at the screening: 2,218/3,472 (73%)**		**Univariate analysis**		**Multivariate analysis**	
	**Total population n = 3472 (%)**	**Odds ratio (95% CI)**	**p-value**	**Odds ratio (95% CI)**	**p-value**
***Age groups***					
**<25**	1430 (41%)	1		1	
**25-34**	1275 (37%)	2.37 (2.00,2.81)	<0.001	1.82 (1.49,2.22)	<0.001
**35+**	767 (22%)	3.18 (2.55,3.95)	<0.001	3.49 (2.71,4.49)	<0.001
***Language spoken***					
**English/other**	191 (6%)	1			
**Zulu**	3281 (94%)	5.47 (4.01,7.43)	<0.001	-	
***Level of education***					
**At least high school**	904 (26%)	1		1	
**Less than high school**	2568 (74%)	1.68 (1.43,1.98)	<0.001	1.26 (1.03,1.53)	0.021
**Religion**					
**Other**	325 (9%	1			
**Christian**	3147 (91%)	1.72 (1.36,2.18)	<0.001	-	
***Age at sexual debut***					
**>16 years**	2073 (60%)	1			
**16 years or younger**	1399 (40%)	1.40 (1.19,1.63)	<0.001	-	
***Marital/cohabitation status***					
**Married/cohabitating**	528 (15%)	1			
**Not married/not cohabitating**	2944(85%)	1.54 (1.25,1.89)	<0.001	-	
**Number of life time sexual partners**					
**1**	854 (25%)	1		1	
**2**	978 (28%)	2.53 (2.10,3.10)	<0.001	1.89 (1.52,2.34)	<0.001
**3**	731 (21%)	3.84 (3.10,4.82)	<0.001	2.30 (1.78,2.96)	<0.001
**4 +**	906 (26%)	5.43 (4.33,6.82)	<0.001	2.48 (1.92,3.20)	<0.001
**Condom used (ever)**					
**Yes**	2438 (71%)	1			
**No**	1034 (29%)	1.03 (0.88,1.22)	0.689	-	
**Parity**					
**None**	512 (15%)	1		1	
**>1**	2960 (85%)	2.31 (1.90,2.80)	<0.001	1.95 (1.54,2.48)	<0.001
***Contraceptive use (any)***					
**Yes**	2692 (78%)	1			
**No**	780 (22%)	1.40 (1.26,1.68)	0.001	-	
**HIV status at the screening**					
**seronegative**	2041 (59%)	1		1	<0.001
**seropositive**	1431 (41%)	7.38 (6.01,9.10)	<0.001	6.31 (5.06,7.88)	
***Trichomonas vaginalis*** **infection at the screening**					
**Negative**	3242 (94%)	1			
**Positive**	216 (6%)	1.04 (0.76,1.43)	0.786	-	
***Chlamydia trachomatis*** **infection at the screening**					
**Negative**	3149 (91%)	1			
**Positive**	309 (9%)	1.00 (0.77,1.30)	0.999	-	
***Neisseria gonorrhoea*** **infection at the screening**					
**Negative**	3375 (97%)	1			
**Positive**	97 (3%)	2.28 (1.29,4.05)	0.005	-	
***Genital signs and symptoms*** ^***1***^					
**Genital epithelial disruption**		1			
**No**	3403 (98%)	1		-	
**Yes**	69 (2%)	1.48 (0.62,3.52)	0.372		
**Genital signs**					
**No**	2986 (86%)	1		-	
**Yes**	486 (14%)	0.98 (0.69,1.39)	0.895		
**Genital discharge**		1			
**No**	2500 (72%)	1		-	
**Yes**	972 (28%)	0.94 (0.72,1.22)	0.628		
**Genital ulcer**					
**No**	3264 (94%)	1		-	
**Yes**	208 (6%)	3.16 (1.73,5.74)	<0.001		
**Bacterial Vaginosis infection**					
**No**	3160 (91%)	1		-	
**Yes**	312 (9%)	1.32 (0.89,1.96)	0.162		

*CI: Confidence Interval.

^1^In vulva, vaginal or cervical.

In the multivariate analyses, older women (25 years and older) were more likely to be HSV-2 sero-positive (OR: 1.82, 95% CI: 1.49, 2.22). Women with a lower level of education were also considered to be more likely to be HSV-2 positive (OR: 1.26 95% CI: 1.03, 1.53). Additionally, having >1 life-time sexual partners (OR: 2.48, 95% 1.92, 3.20) parity >1 (OR: 1.95 95% CI: 1.92, 3.20) and being HIV positive (OR: 6.31, 95% CI 5.06, 7.88) were all significantly associated with HSV-2 infection.

In this study, the prevalence of HSV-2 was estimated at 73% with 41% of the women also co-infected with HIV. Behavioural characteristics such as: a high number of life-time sexual partners [12-15], older age [9,10,16,17], parity >1 [15,17] and a low level of education [17] were contributory factors. Biologically, being HIV positive [9,15,16,18], was associated with the high HSV-2 sero-prevalence seen in the study population. The data reported in this study is consistent with previous studies [9,10,16,17].

Consistent with other studies, older age is associated with HSV-2 infection [9,10,16,17]. The prevalence of HSV-2 infection in women with > 4 life-time sexual partners was 2-fold higher when compared with women with just one sexual partner. This finding is strongly supported by previous studies conducted [12-15]. Parity was also strongly associated with HSV-2 infection. Women with a previous pregnancy had a 2-fold higher prevalence for HSV-2 infection when compared to women that were nulliparous. Currently there is limited published literature that explains the association between parity and HSV-2 infections. Older age and parity are probably surrogate measures of cumulative sexual exposure. However, post-partum susceptibility to HSV-2 will be best explained in a prospective analysis. Our findings show that HSV-2 infections was higher in women with a lower level of education (less than high-school education) as compared to women with high-school education. This is consistent with the findings of Uribe-Salas et al. [17].

In this study we found that women that were already HIV positive had 6-fold greater odds of being co-infected with HSV-2 than women that were HIV sero-negative. HIV has been shown to enhance the genital shedding of HSV-2, despite the absence of clinical lesions [19]. In Durban, the MIRA study was undertaken from a peri-urban clinic in Umkomaas and a less urbanised clinic in Botha’s Hill. The women that visited the clinics were at higher risk for HIV and STIs since they were residing in areas termed “hotspots” with excessively high HIV prevalence rates ranging from 39-56%. Diagnosis of HSV-2 was also more common in the hotspots [20].

In this study, we used a cut-off value of 1.1 which has been previously shown to have reduced sensitivity and specificity in detecting HSV-2 antibodies [21]. Despite this limitation, this study was able to identify factors that have been strongly associated with prevalent HSV-2 infections and the factors identified in this study are consistent with previous published findings.The high sero-prevalence of HSV-2 in the studied population is of great public health importance since this high risk population could contribute to the transmission of future infections particularly HIV. This study provides insight into the factors that contribute to prevalent HSV-2 infection. The identification of risk factors for HSV-2 infection could aid in the development and implementation of personalized prevention messages for this infection.

We acknowledge the following limitations of our study: the population studied here volunteered to participate in an HIV/STI prevention clinical trial and therefore could be assumed to be at being at more risk for HIV/STIs. Sexual behaviour data was collected by self-report and therefore introduces the concept of biasness. The HerpeSelect™ ELISA IgG has been shown to have a reduced sensitivity and specificity in populations of high HIV prevalence which could have affected interpretation of the results [21]. And finally, laboratory tests that identified exactly which pathogens caused the ulcers as well as further characterization of the clinical symptoms that fell in the category genital signs would also have made useful discussion points. And finally, due to the cross-sectional design of this study, temporal relationships could not be established.
